# Tobacco Product Use Among High School Students — Youth Risk Behavior Survey, United States, 2019

**DOI:** 10.15585/mmwr.su6901a7

**Published:** 2020-08-21

**Authors:** MeLisa R. Creamer, Sherry Everett Jones, Andrea S. Gentzke, Ahmed Jamal, Brian A. King

**Affiliations:** ^1^Office on Smoking and Health, National Center for Chronic Disease Prevention and Health Promotion, CDC; ^2^Division of Adolescent and School Health, National Center for HIV/AIDS, Viral Hepatitis, STD, and TB Prevention, CDC

## Abstract

Tobacco product use is the leading cause of preventable disease, disability, and death in the United States. This report used data from the 2019 Youth Risk Behavior Survey to assess the following among U.S. high school students: ever use of cigarettes and electronic vapor products, current use (≥1 day during the 30 days before the survey) of tobacco products, frequent use (≥20 days during the 30 days before the survey) among current users of tobacco products, trends in use over time, and usual source of electronic vapor products among current electronic vapor product users. In 2019, a total of 50.1% of U.S. high school students had ever used electronic vapor products, and 24.1% had ever tried cigarette smoking. Current electronic vapor product use was 32.7%, current cigarette smoking was 6.0%, current cigar smoking was 5.7%, and current smokeless tobacco use was 3.8%. Approximately 36.5% of students were current users of any tobacco product, and 8.2% were current users of two or more tobacco products. Frequent use among users of individual products was 32.6% for electronic vapor products, 28.5% for smokeless tobacco, 22.2% for cigarettes, and 18.4% for cigars. Among current electronic vapor product users who were aged ≤17 years, the most commonly reported source was borrowing them from someone else (42.8%). Significant decreases occurred in current cigarette smoking (1991: 27.5%; 2019: 6.0%), cigar smoking (1997: 22.0%; 2019: 5.7%), and smokeless tobacco use (2017: 5.5%; 2019: 3.8%). However, significant increases occurred in current electronic vapor product use (2015: 24.1%; 2019: 32.7%) and any tobacco product use (2017: 19.5%; 2019: 36.5%). Although current cigarette smoking, cigar smoking, and smokeless tobacco use has decreased among high school students, the increased prevalence of electronic vapor product use among youths is concerning. Continued surveillance for all tobacco product use is warranted for guiding and evaluating public health policy at the local, state, tribal, and national levels.

## Introduction

Smoking is the leading cause of preventable premature disease and death in the United States (*1*). An estimated 88% of adult daily cigarette smokers report first trying cigarette smoking before age 18 years (*2*). Previous reports indicate decreases in current cigarette smoking (i.e., use during the 30 days before the survey) among U.S. high school students from a high of 36.4% in 1997 to 8.8% in 2017 (*3*). However, there are a variety of tobacco products, including smokeless tobacco products, cigars, and most recently, electronic vapor products (e.g., e-cigarettes).

Electronic vapor products have evolved since entering the U.S. marketplace in 2007. Initial products were disposable, resembled the size and shape of conventional cigarettes, and used free-base nicotine; however, newer products are rechargeable, resemble common objects (e.g., USB flash drives), and typically deliver nicotine salts (*4*,*5*), which allow higher levels of nicotine to be inhaled more easily by the user (*6*). Sales of these newer generation, or “pod-mod,” products have increased in the United States during recent years. For example, sales of JUUL, the most commonly sold e-cigarette in the United States since December 2017, increased approximately 600% during 2016–2017 from 2.2 million unit sales to 16.2 million unit sales (*7*). By December 2018, JUUL accounted for an estimated 76% of the $322.1 million total e-cigarettes sales that occurred that month in the United States (*8*). The popularity of these electronic vapor products among youths is likely the result of multiple factors, including advertising exposure, availability of youth-appealing flavors, curiosity, and social exposure through friends and others (*4*–*6*).

In 2014, prevalence of electronic vapor product use among high school students surpassed prevalence of cigarette smoking (*9*), and according to data from the 2017 Youth Risk Behavior Survey (YRBS), 13.2% of high school students had used electronic vapor products during the previous 30 days (*3*). These findings align with increases in use observed in other national surveys of youth in the United States. For example, according to the National Youth Tobacco Survey (NYTS), current electronic vapor product use among high school students increased 78% (11.7% to 20.8%) during 2017–2018 (*9*).

Youth use of tobacco products in any form is unsafe (*1*,*4*). Cigarette smoking harms nearly every organ in the body, and smokeless tobacco product use is associated with multiple health risks, including cancers of the mouth (*1*). Moreover, the aerosol in electronic vapor products can contain harmful ingredients, including heavy metals, ultrafine particles, and nicotine (*4*). Nicotine is highly addictive, can harm the developing adolescent brain, and can prime the brain for addiction to other drugs (*4*,*10*). In addition, a growing body of scientific literature suggests that youths who use e-cigarettes are more likely to smoke conventional cigarettes in the future (*4*,*10*).

Surveillance for tobacco product use among youths is crucial for guiding and evaluating tobacco control strategies at local, state, tribal, and national levels. This report presents the latest data from the 2019 YRBS to assess the following among U.S. high school students: ever use of cigarettes and electronic vapor products; current use (≥1 day during the 30 days before the survey) of tobacco products (electronic vapor products, cigarettes, cigars [cigars/cigarillos/little cigars], smokeless tobacco [chewing tobacco, snuff, dip, snus, or dissolvable tobacco products], any tobacco product, and two or more products); frequent use (≥20 days during the 30 days before the survey) of tobacco products among current users of those products; trends in tobacco product use over time; and usual source of obtaining electronic vapor products among current electronic vapor product users. 

## Methods

### Data Source

This report includes data from the 1991–2019 cycles of CDC’s national YRBS, a cross-sectional, school-based survey conducted biennially since 1991. Each survey year, CDC collects data from a nationally representative sample of public and private school students in grades 9–12 in the 50 U.S. states and the District of Columbia. Additional information about YRBS sampling, data collection, response rates, and processing is available in the overview report for this supplement (*11*). The prevalence estimates for all tobacco product use questions for the overall study population and by sex, race/ethnicity, grade, and sexual orientation are available at https://nccd.cdc.gov/youthonline/App/Default.aspx. The full YRBS questionnaire is available at https://www.cdc.gov/healthyyouth/data/yrbs/pdf/2019/2019_YRBS-National-HS-Questionnaire.pdf.

### Measures

Ever use, which was defined as having used the product at least one time during their lifetime, was assessed for two distinct tobacco products: cigarettes and electronic vapor products. Ever cigarette smoking was assessed by the question, “Have you ever tried cigarette smoking, even one or two puffs?” Ever electronic vapor product use was assessed by the question, “Have you ever used an electronic vapor product?” with a preamble that read, “The next 3 questions ask about electronic vapor products, such as JUUL, Vuse, MarkTen, and blu. Electronic vapor products include e-cigarettes, vapes, vape pens, e-cigars, e-hookahs, hookah pens, and mods.”

Current use (≥1 day during the 30 days before the survey) was assessed for four tobacco products: 1) current electronic vapor product use was assessed by the question, “During the past 30 days, on how many days did you use an electronic vapor product?” 2) current cigarette smoking was assessed by the question, “During the past 30 days, on how many days did you smoke cigarettes?” 3) current cigar smoking was assessed by the question, “During the past 30 days, on how many days did you smoke cigars, cigarillos, or little cigars?” and 4) current smokeless tobacco use was assessed by the question, “During the past 30 days, on how many days did you use chewing tobacco, snuff, dip, snus, or dissolvable tobacco products, such as Copenhagen, Grizzly, Skoal, or Camel Snus? (Do not count any electronic vapor products.)” Response options for each of the four questions were 0 days, 1–2 days, 3–5 days, 6–9 days, 10–19 days, 20–29 days, and all 30 days. Among current users of each individual product, frequent use was also calculated. Frequent use was defined as having used the respective product on ≥20 days during the 30 days before the survey.

Two composite measures were also investigated in this analysis. Any current tobacco product use was defined as any use of electronic vapor products, cigarettes, cigars, or smokeless tobacco during the 30 days before the survey. Use of two or more products was defined as current use of two or more of the four assessed tobacco products.

Respondents also were asked how they usually obtained electronic vapor products by the question (referred to as source hereinafter), “During the past 30 days, how did you usually get your own electronic vapor products? (Select only one response.)” Response options were as follows: I did not use any electronic vapor products during the past 30 days; I bought them in a store such as a convenience store, supermarket, discount store, gas station, or vape store; I got them on the Internet; I gave someone else money to buy them for me; I borrowed them from someone else; a person who can legally buy these products gave them to me; I took them from a store or another person; or I got them some other way. Analysis of this variable was limited to current electronic vapor product users.

The demographic characteristics of students analyzed for this report included sex (female or male), grade (9, 10, 11, or 12), age (≤15 years, 16 or 17 years, or ≥18 years), and sexual identity (heterosexual; lesbian, gay, or bisexual; or not sure). In addition, students were classified into four racial/ethnic categories: non-Hispanic white (white); non-Hispanic black (black); Hispanic or Latino of any race (Hispanic); and other or multiple races (non-Hispanic). The numbers of students in the other or multiple racial/ethnic groups were too small to produce statistically stable estimates; therefore, those data are not presented as a separate group in this report but were retained in the overall analytic sample.

### Analysis

Prevalence of use for each respective tobacco product was estimated for all years for which data were available. For 2019, statistically significant pairwise differences by sex, grade, race/ethnicity, age, and sexual identity were determined for each of the assessed tobacco product use behaviors by using *t-*tests. For each tobacco product, changes in prevalence were compared for 2017 and 2019 by using *t*-tests. In addition, *t*-tests were used to compare how students who were ≤17 years and ≥18 years usually obtained their electronic vapor products; these age groups were used because age 18 years was the federal legal age of sale for tobacco products at the time of the survey. Prevalence estimates were considered statistically different if the p value was <0.05.

To identify temporal trends, logistic regression analyses were used to model linear and quadratic time effects while controlling for sex, grade, and race/ethnicity. Linear time effects were analyzed for current electronic vapor products use (2015–2019), and both linear and quadratic time effects were analyzed for current cigarette smoking (1991–2019) and current cigar smoking (1997–2019). Because of substantial changes in the question wording for smokeless tobacco products in 2017, trends were not assessed for smokeless tobacco. Additional information about the methods used to conduct YRBS trend analyses are provided in the overview report of this supplement (*11*).

## Results

Among U.S. high school students in 2019, a total of 50.1% (95% confidence interval [CI]: 48.1–52.2) had ever used electronic vapor products, and 24.1% (CI: 21.3–27.0) had ever tried cigarette smoking (data not shown). Prevalence of current use was 32.7% for electronic vapor products, 6.0% for cigarettes, 5.7% for cigars, and 3.8% for smokeless tobacco. In addition, 36.5% of students had currently used any tobacco products, and 8.2% had currently used two or more tobacco products (Table 1).

**TABLE 1:** Percentage of high school students who were current tobacco users, by selected characteristics and type of tobacco product — Youth Risk Behavior Survey, United States, 2019

Characteristic	Electronic vapor products*	Cigarettes^†^	Cigars^§^	Smokeless tobacco^¶^	Any tobacco product**	≥2 products^††^
	% (95% CI)	% (95% CI)	% (95% CI)	% (95% CI)	% (95% CI)	% (95% CI)
**Total**	**32.7 (30.7–34.8)**	**6.0 (5.0–7.2)**	**5.7 (4.8–6.7)**	**3.8 (3.2–4.6)**	**36.5 (33.6–39.5)**	**8.2 (7.0–9.5)**
**Sex^§§^**						
Male	32.0 (29.7–34.3)	6.9 (5.7–8.4)	7.4 (6.4–8.6)	5.8 (4.7–7.1)	36.3 (33.3–39.3)	10.4 (9.0–11.9)
Female	33.5 (30.9–36.1)	4.9 (3.8–6.4)	3.8 (2.8–5.1)	1.6 (1.2–2.1)	36.6 (33.1–40.2)	5.8 (4.5–7.5)
**Grade^¶¶^**						
9	25.0 (22.8–27.4)	3.8 (2.8–5.1)	3.8 (2.7–5.2)	2.0 (1.4–3.0)	27.7 (24.8–30.9)	5.3 (4.2–6.6)
10	30.5 (27.3–33.8)	5.2 (3.9–6.9)	4.7 (3.5–6.2)	3.6 (2.6–5.0)	34.3 (30.3–38.6)	7.3 (5.6–9.6)
11	35.9 (32.3–39.8)	5.9 (4.5–7.7)	6.0 (4.6–7.8)	3.9 (3.0–5.1)	39.8 (35.7–44.1)	8.4 (6.7–10.4)
12	40.4 (37.5–43.4)	9.0 (7.6–10.7)	8.5 (6.9–10.4)	5.5 (4.3–7.1)	45.0 (41.3–48.7)	11.9 (10.3–13.7)
Race/Ethnicity***						
Black, non-Hispanic	19.7 (16.9–22.8)	3.3 (2.3–4.6)	5.3 (4.1–6.8)	2.8 (1.8–4.4)	24.7 (21.3–28.4)	4.8 (3.7–6.2)
Hispanic	31.2 (28.6–33.8)	6.0 (4.3–8.4)	6.1 (4.7–8.0)	3.1 (2.3–4.3)	33.8 (31.1–36.7)	7.9 (6.2–10.0)
White, non-Hispanic	38.3 (36.0–40.7)	6.7 (5.3–8.4)	5.9 (4.7–7.4)	4.4 (3.3–5.7)	42.0 (38.3–45.9)	9.5 (7.8–11.5)
**Age group (yrs)** ^†††^						
≤15	25.9 (24.1–27.9)	4.2 (3.2–5.4)	4.2 (3.1–5.6)	2.7 (2.0–3.8)	29.1 (26.2–32.1)	5.8 (4.6–7.2)
16 or 17	35.2 (32.3–38.3)	6.0 (4.8–7.4)	5.7 (4.5–7.0)	3.7 (3.0–4.6)	38.8 (35.2–42.4)	8.4 (6.8–10.1)
≥18	42.8 (39.0–46.7)	10.9 (8.6–13.6)	10.2 (8.1–12.7)	7.2 (5.5–9.2)	49.1 (44.9–53.4)	14.2 (12.0–16.7)
**Sexual identity^§§§^**						
Heterosexual	32.8 (30.5–35.2)	5.2 (4.3–6.3)	5.2 (4.4–6.1)	3.7 (3.1–4.4)	36.1 (33.1–39.2)	7.8 (6.7–9.0)
Lesbian, gay, or bisexual	34.1 (30.8–37.6)	10.4 (7.8–13.7)	8.1 (5.9–11.1)	3.2 (2.0–5.2)	40.3 (36.2–44.4)	10.4 (8.0–13.5)
Not sure	24.9 (19.8–30.7)	7.4 (4.8–11.3)	7.2 (4.3–12.0)	5.5 (3.1–9.5)	30.0 (23.3–37.6)	8.1 (5.4–11.9)

**Abbreviation:** CI = confidence interval.

* Percentage of students who used an electronic vapor product, including e-cigarettes, e-cigars, e-pipes, vape pipes, vaping pens, e-hookahs, and hookah pens (e.g., blu, NJOY, Vuse, MarkTen, Logic, Vapin Plus, eGo, and Halo), on ≥1 day during the 30 days before the survey.

^†^ Percentage of students who smoked cigarettes on ≥1 day during the 30 days before the survey.

^§^ Percentage of students who smoked cigars, cigarillos, or little cigars on ≥1 day during the 30 days before the survey.

^¶^ Percentage of students who used smokeless tobacco, including chewing tobacco, snuff, dip, snus, or dissolvable tobacco products (e.g., Red Man, Levi Garrett, Beechnut, Skoal, Skoal Bandits, Copenhagen, Camel Snus, Marlboro Snus, General Snus, Ariva, Stonewall, or Camel Orbs), but not including any electronic vapor products, on ≥1 day during the 30 days before the survey.

** Percentage of students who smoked cigarettes or cigars or used smokeless tobacco or an electronic vapor product, on ≥1 day during the 30 days before the survey.

^††^ Percentage of students who used ≥2 of the following tobacco products: cigarettes, cigars (cigars, cigarillos, or little cigars), an electronic vapor product, or smokeless tobacco, on ≥1 day during the 30 days before the survey.

^§§^ Sex pairwise comparisons assessed by *t*-test (p<0.05): for cigarettes, cigars, smokeless tobacco, and ≥2 products, male students were significantly different (p<0.05) from female students.

^¶¶^ Grade pairwise comparisons assessed by *t*-test (p<0.05): for electronic vapor products and any tobacco product: all pairwise comparisons were significantly different (p<0.05); for cigarettes, cigars, and ≥2 products: 12th grade was significantly different (p<0.05) than 9th, 10th, and 11th grades; 11th grade was significantly different (p<0.05) than 9th grade; for smokeless tobacco: 12th grade was significantly different (p<0.05) than 9th, 10th, and 11th grades; 10th and 11th grades were significantly different (p<0.05) than 9th grade.

*** Race/ethnicity pairwise comparisons assessed by *t*-test (p<0.05): for electronic vapor products and any tobacco product: all pairwise comparisons were significantly different (p<0.05); for cigarettes and ≥2 products: white and Hispanic were significantly different (p<0.05) than black.

^†††^ Age pairwise comparisons assessed by *t*-test (p<0.05): for electronic vapor products, cigarettes, smokeless tobacco, any tobacco product, and ≥2 products: all pairwise comparisons were significantly different (p<0.05); for cigars: ≥18 years was significantly different (p<0.05) than 16–17 years and ≤15 years.

^§§§^ Sexual identity pairwise comparisons assessed by *t*-test (p<0.05): for electronic vapor products: heterosexual and lesbian, gay, or bisexual were significantly different (p<0.05) than not-sure students; for cigarettes, cigars, and ≥2 products: lesbian, gay, or bisexual was significantly different (p<0.05) than heterosexual; for any tobacco product: lesbian, gay, or bisexual was significantly different (p<0.05) than heterosexual and not-sure students.

Prevalence of tobacco product use varied by demographic groups, with current use of cigarettes, cigars, smokeless tobacco, and two or more tobacco products being higher among male students than female students. Although differences in tobacco product use varied by grade, prevalence of current use of each individual product, any tobacco product, and two or more tobacco products was higher among 12th-grade students than 9th-grade students. Prevalence of current use of electronic vapor products, cigarettes, any tobacco product, and two or more tobacco products was higher among white and Hispanic students than black students, and the prevalence of electronic vapor products and any tobacco product use was higher among white than Hispanic students. Prevalence of current cigar use was higher among students aged ≥18 years than those aged 16 or 17 years and those aged ≤15 years. For all other individual products, any tobacco product, and two or more tobacco products, prevalence increased in each age category. Among sexual identity groups, prevalence of electronic vapor product use was higher among heterosexual students and lesbian, gay, or bisexual students than not-sure students. Prevalence of current use of cigarettes, cigars, any tobacco product, and two or more tobacco products was higher among lesbian, gay, or bisexual students than heterosexual students. Finally, the prevalence of any tobacco product use was higher among lesbian, gay, or bisexual students than not-sure students.

**FIGURE 1:**
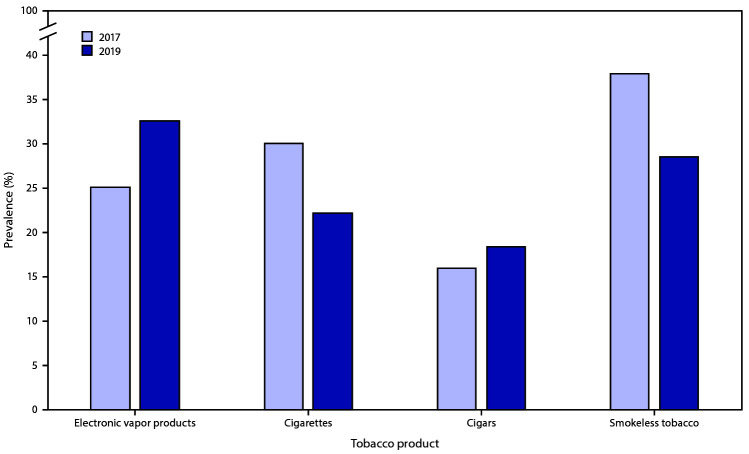
Prevalence of frequent tobacco use* among current users, by type of tobacco product^†^ — Youth Risk Behavior Survey, United States, 2017 and 2019^§^ * Frequent use was defined as use on ≥20 days during the 30 days before the survey. ^†^ Frequent use was assessed among respondents who reported current use (on ≥1 day during the 30 days before the survey) of each tobacco product. In 2017, among the 13.2% of students nationwide who used electronic vapor products on ≥1 day during the 30 days before the survey; among the 8.8% of students nationwide who smoked cigarettes on ≥1 day during the 30 days before the survey; among the 8.0% of students nationwide who smoked cigars on ≥1 day during the 30 days before the survey; among the 5.5% of students nationwide who used smokeless tobacco on ≥1 day during the 30 days before the survey. In 2019, among the 32.7% of students nationwide who used electronic vapor products on ≥1 day during the 30 days before the survey; among the 6.0% of students nationwide who smoked cigarettes on ≥1 day during the 30 days before the survey; among the 5.7% of students nationwide who smoked cigars on ≥1 day during the 30 days before the survey; among the 3.8% of students nationwide who used smokeless tobacco on ≥1 day during the 30 days before the survey. ^§^ Differences from 2017 to 2019 were assessed by *t*-test (p<0.05): A significant increase occurred in frequent use of electronic vapor products; a significant decrease occurred in frequent use of cigarettes; and no change occurred in frequent use of cigars/cigarillos/little cigars and smokeless tobacco.

In 2019, among the 32.7% of current electronic vapor product users, 32.6% were frequent users; among the 5.7% current cigarette smokers, 22.2% were frequent users; among the 3.8% current cigar smokers, 18.4% were frequent users; and among the 6.0% current smokeless tobacco product users, 28.5% were frequent users. From 2017 to 2019, among current electronic vapor product users, a significant increase occurred in frequent use (from 25.1% to 32.6%), and among current cigarette smokers, a significant decrease occurred in frequent use (from 30.0% in 2017 to 22.2% in 2019) (Figure 1). No significant changes in frequent use of smokeless tobacco or cigars were observed among users of these products from 2017 to 2019.

The usual source of electronic vapor products among current users varied by age (Table 2). Among current electronic vapor product users who were aged ≤17 years, the most commonly reported usual source of electronic vapor products was borrowing them from someone else (42.8%). Among those aged ≥18 years, the most commonly reported source was buying them in a store (56.4%). Compared with students aged ≤17 years, a higher prevalence of students aged ≥18 years usually bought electronic vapor products in a store. In contrast, compared with older students, a higher prevalence of students aged ≤17 years got them on the Internet, gave someone else money to buy them, borrowed them from someone else, got them from a person who could legally buy them, or got them some other way.

**TABLE 2:** Usual source* of obtaining electronic vapor products among current electronic vapor product users,^†^ by age — Youth Risk Behavior Survey, United States, 2019

Usual source	Age group^§^	
	≥18 yrs	≤17 yrs
	% (95% CI)	% (95% CI)
Bought them in a store (e.g., a convenience store, supermarket, discount store, gas station, or vape store)	56.4 (51.0–1.6)	8.1 (6.8–9.6)
Got them on the Internet	1.8 (0.9–3.4)	3.6 (2.8–4.6)
Gave someone else money to buy them for me	3.1 (1.5–6.1)	21.3 (19.5–23.2)
Borrowed them from someone else	27.5 (23.4–32.0)	42.8 (40.2–45.4)
A person who can legally buy these products gave them to me	3.9 (2.4–6.3)	11.1 (9.9–12.3)
Took them from a store or another person	2.0 (0.8–5.0)	1.6 (1.1–2.4)
Got them some other way	5.4 (3.3–8.8)	11.6 (10.1–13.4)

**Abbreviation:** CI = confidence interval.

* Students were limited to selecting only one response.

^†^ Including e-cigarettes, e-cigars, e-pipes, vape pipes, vaping pens, e-hookahs, or hookah pens (e.g., blu, NJOY, Vuse, MarkTen, Logic, Vapin Plus, eGo, and Halo) among students who used electronic vapor products during the 30 days before the survey.

^§^ Comparisons between age groups were assessed by *t*-test (p<0.05). All comparisons were statistically different with the exception of “took them from a store or another person.”

**FIGURE 2:**
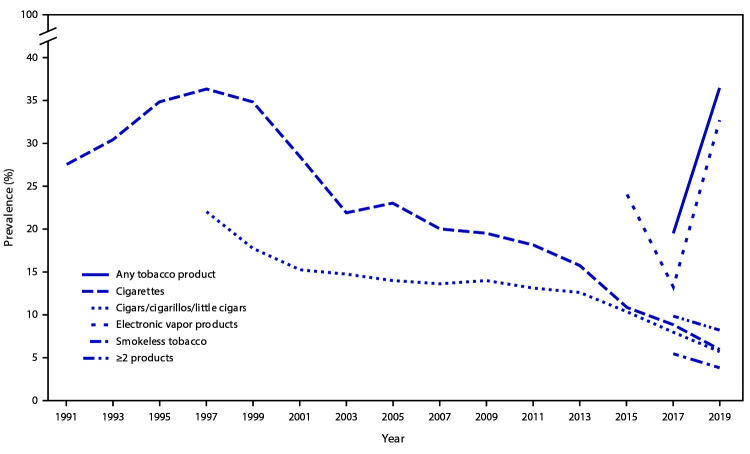
Prevalence of current tobacco product use, by year — Youth Risk Behavior Survey, United States, 1991–2019* * Logistic regression analyses were used to model linear and quadratic time effects while controlling for sex, grade, and race/ethnicity. Electronic vapor products: significant linear increase (2015–2019); cigarettes: significant linear decrease (1991–2019); significant quadratic trend: increase during 1991–1997, decrease during 1997–2019; cigars/cigarillos/little cigars: significant linear decrease (1997–2019); significant quadratic trend: decrease 1997–2013; decrease 2013–2019 (different rate of decrease). Differences from 2017 to 2019 were assessed by *t*-test (p<0.05): A significant increase occurred in use of electronic vapor products and any tobacco product; a significant decrease occurred in use of cigarettes, cigars/cigarillos/little cigars and smokeless tobacco; and no change occurred in use of ≥2 products.

Trend analyses indicated that during 2015–2019, a significant linear increase occurred in prevalence of current electronic vapor products use (from 24.1% to 32.7%) (Figure 2). Trend analyses also indicated that during 1991–2019, a significant linear decrease in current cigarette smoking was observed (from 27.5% to 6.0%). A significant quadratic trend in cigarette smoking also was identified: a 6-year increase in prevalence (from 27.5% in 1991 to 36.4% in 1997) was followed by a 22-year decrease (from 36.4% in 1997 to 6.0% in 2019). Additionally, during 1997–2019, a significant linear decrease (from 22.0% to 5.7%) occurred in the overall prevalence of current cigar smoking. A significant quadratic trend also was identified: a 16-year decrease in prevalence (from 22.0% in 1997 to 12.6% in 2013) was followed by another 6-year decrease, but at a different rate of decrease (from 12.6% in 2013 to 5.7% in 2019).

During 2017–2019, a significant increase occurred in current electronic vapor products use (from 13.2% to 32.7%) and any tobacco product use (from 19.5% to 36.5%). During 2017–2019, significant decreases were observed in current cigarette smoking (from 8.8% to 6.0%), current cigar smoking (from 8.0% to 5.7%), and current smokeless tobacco use (from 5.5% to 3.8%). No change occurred in use of two or more tobacco products during 2017–2019.

## Discussion

In 2019, a total of 36.5% of high school students currently used any tobacco product, with electronic vapor products being the most commonly used product. This reflects an increase in use of electronic vapor products from 2017 to 2019, findings that are consistent with those from other national surveillance systems, including NYTS (*9*,*12*) and Monitoring the Future (*13*). For example, NYTS results demonstrated that, among high school students, e-cigarette use increased from 11.7% in 2017 to 27.5% in 2019 (*9*,*12*). These increases align with the increasing popularity of newer electronic vapor product devices, including JUUL (*7*). The dramatic increase in electronic vapor product use among high school students has led to increases in overall tobacco product use among U.S. youths, erasing gains made in previous years and leading the U.S. Surgeon General to declare youth e-cigarette use an epidemic in the United States (*10*).

Use of any tobacco product among youth is unsafe, regardless of frequency of use or number of products used. Although the 2019 national YRBS results indicate that most current youth tobacco product users are infrequent users, variations exist by product; for example, frequent use ranged from 18.4% for cigars to 32.6% for electronic vapor products. In addition, these results indicate that frequent use of electronic vapor products increased during 2017–2019; whereas frequent use of other products decreased or did not change. Even infrequent tobacco product use, particularly cigarette smoking, is predictive of progression to daily smoking (*14*). Nearly all tobacco products include nicotine, and even infrequent use of tobacco products has been linked to symptoms of nicotine dependence (*15*). Further, 8.2% of high school students currently used two or more tobacco products in 2019. Multiple tobacco product use is associated with substance use disorders (*16*) and might increase nicotine exposure and risk for nicotine dependence (*15*).

In 2019, electronic vapor product users aged ≤17 years usually obtained their products from social sources (e.g., by borrowing them from someone). This is consistent with results from both the Population Assessment of Tobacco and Health Study and NYTS, which also determined that social sources were the most common way for adolescents to obtain electronic vapor products (*17*,*18*). These social sources might include older students who are of legal age for purchasing the products in their state or community. In 2016, electronic vapor products were deemed to be tobacco products under the Family Smoking Prevention Tobacco Control Act (https://www.federalregister.gov/documents/2016/05/10/2016-10685/deeming-tobacco-products-to-be-subject-to-the-federal-food-drug-and-cosmetic-act-as-amended-by-the), thus setting the federal minimum purchase age for electronic vapor products at 18 years. However, on December 20, 2019, federal legislation increased the minimum age of sales of tobacco products from 18 to 21 years nationwide; the law does not preempt more stringent state or local age of sale laws (https://www.fda.gov/tobacco-products/retail-sales-tobacco-products/selling-tobacco-products-retail-stores). Before this federal law, 19 states, the District of Columbia, Guam, and Palau had enacted laws that increased the age of sale for tobacco products to 21 years, including 13 laws enacted during 2019 (*19*). Such laws might limit the ability for high school students to obtain tobacco products from their peers, including those older students who were of legal age to purchase these products in their state or community before the law’s implementation.

Multiple factors continue to promote and influence tobacco product use among youths, including exposure to tobacco product advertising and imagery through media, as well as the availability of flavored tobacco products. The sustained and comprehensive implementation of population-based strategies, in coordination with the regulation of tobacco products by the U.S. Food and Drug Administration (FDA), can reduce all forms of tobacco product use and initiation among U.S. youths. Such strategies include increasing the price of tobacco products, implementing comprehensive smoke-free policies, implementing advertising and promotion restrictions and national antitobacco public education media campaigns, restricting youth access to flavored tobacco products, and implementing policies that increase the minimum age of purchase for tobacco products to 21 years (*1*,*2*,*4*,*10*). In addition to population-level policies for preventing and reducing initiation of tobacco product use among youths, tools from the National Cancer Institute (e.g., https://teen.smokefree.gov) and the Truth Initiative (e.g., https://truthinitiative.org/thisisquitting) provide resources to help youth quit tobacco product use.

## Limitations

Limitations for YRBS overall are available in the overview report of this supplement (*11*). This report is subject to at least three additional limitations. First, changes in question wording for smokeless tobacco use in 2017 prohibit comparability with previous years’ data and long-term trend analyses for prevalence of smokeless tobacco use, any tobacco product use, and use of two or more tobacco products. Second, the question addressing how students usually obtained electronic vapor products requires that respondents select only one response, although they might have obtained these products through multiple sources; therefore, the full scope of the sources students use to access these products might not have been addressed. Finally, the questions related to electronic vapor products and cigars do not specifically exclude the possibility of marijuana use in either product (e.g., blunt use).

## Conclusion

Although current use of cigarettes, cigars, and smokeless tobacco among U.S. high school students has decreased, tobacco product usage has evolved, and the increasing prevalence of electronic vapor product use among youths during recent years is concerning. Implementing evidence-based tobacco control strategies, combined with FDA’s regulatory efforts, is important for preventing and reducing all forms of tobacco product use among youths. In addition, continued surveillance of all tobacco products is warranted for guiding and evaluating public health policy at the local, state, tribal, and national levels.
