# *Erratum:* Vol. 69, No. Suppl. 1

**DOI:** 10.15585/mmwr.mm6940a4

**Published:** 2020-10-09

In the *MMWR Supplement* report “Tobacco Product Use Among High School Students — Youth Risk Behavior Survey, United States, 2019,” errors occurred on page 58. In the second column, the first sentence of the second full paragraph should have read “In 2019, among the 32.7% of current electronic vapor product users, 32.6% were frequent users; among the **6.0%** current cigarette smokers, 22.2% were frequent users; among the **5.7%** current cigar smokers, 18.4% were frequent users; and among the **3.8%** current smokeless tobacco product users, 28.5% were frequent users.”

